# Three-dimensional finite element analysis of the effect of alveolar cleft bone graft on the maxillofacial biomechanical stabilities of unilateral complete cleft lip and palate

**DOI:** 10.1186/s12938-022-01000-y

**Published:** 2022-05-20

**Authors:** Tao Tian, Han-yao Huang, Wei Wang, Bing Shi, Qian Zheng, Cheng-hao Li

**Affiliations:** 1 West China School of Stomatology, Sichuan University, Chengdu, 610041 Sichuan Province The People’s Republic of China; 2 West China Hospital of Stomatology, Sichuan University, Chengdu, 610041 Sichuan Province The People’s Republic of China; 3 Urumql DW Innovation InfoTech Co., Ltd., Urumqi, 830000 Xinjiang Uygur Autonomous Region The People’s Republic of China

**Keywords:** Unilateral complete cleft lip and palate, Alveolar cleft bone graft, Maxillofacial bone, Maxillofacial bone suture, Equivalent stress, Equivalent strain

## Abstract

**Background:**

The objective is to clarify the effect of alveolar cleft bone graft on maxillofacial biomechanical stabilities, the key areas when bone grafting and in which should be supplemented with bone graft once bone resorption occurred in UCCLP (unilateral complete cleft lip and palate).

**Methods:**

Maxillofacial CAD (computer aided design) models of non-bone graft and full maxilla cleft, full alveolar cleft bone graft, bone graft in other sites of the alveolar cleft were acquired by processing the UCCLP maxillofacial CT data in three-dimensional modeling software. The maxillofacial bone EQV (equivalent) stresses and bone suture EQV strains under occlusal states were obtained in the finite element analysis software.

**Results:**

Under corresponding occlusal states, the EQV stresses of maxilla, pterygoid process of sphenoid bone on the corresponding side and anterior alveolar arch on the non-cleft side were higher than other maxillofacial bones, the EQV strains of nasomaxillary, zygomaticomaxillary and pterygomaxillary suture on the corresponding side were higher than other maxillofacial bone sutures. The mean EQV strains of nasal raphe, the maximum EQV stresses of posterior alveolar arch on the non-cleft side, the mean and maximum EQV strains of nasomaxillary suture on the non-cleft side in full alveolar cleft bone graft model were all significantly lower than those in non-bone graft model. The mean EQV stresses of bilateral anterior alveolar arches, the maximum EQV stresses of maxilla and its alveolar arch on the cleft side in the model with bone graft in lower 1/3 of the alveolar cleft were significantly higher than those in full alveolar cleft bone graft model.

**Conclusions:**

For UCCLP, bilateral maxillae, pterygoid processes of sphenoid bones and bilateral nasomaxillary, zygomaticomaxillary, pterygomaxillary sutures, anterior alveolar arch on the non-cleft side are the main occlusal load-bearing structures before and after alveolar cleft bone graft. Alveolar cleft bone graft mainly affects biomechanical stabilities of nasal raphe and posterior alveolar arch, nasomaxillary suture on the non-cleft side. The areas near nasal floor and in the middle of the alveolar cleft are the key sites when bone grafting, and should be supplemented with bone graft when the bone resorbed in these areas.

**Supplementary Information:**

The online version contains supplementary material available at 10.1186/s12938-022-01000-y.

## Background

Based on epidemiological statistics, Nagase et al. [1] found UCCLP (unilateral complete cleft lip and palate) to be the most prevalent type of cleft lip and palate. For patients with UCCLP, the two parts of the maxilla divided by the cleft are also different [2],the asymmetry of the nasomaxillary complexes [3, 4] and the congenital sagittal asymmetric defect of the maxilla with collapsed bone segment deformity [5] on the cleft side are the common clinical manifestations. According to the summary by Janovica et al. [6], the occlusal forces of the dentition formed the specific bony conduction trajectories along the thickened buttresses of the maxillofacial bones, comprising a total of 7 vertical buttresses (bilateral nasomaxillary, zygomaticomaxillary, pterygomaxillary buttresses and the median sagittal buttress) and 3 horizontal buttresses (bilateral prefrontal, zygomatic and maxillary buttresses) to transmit the majority of occlusal loads. The photoelastic technique also revealed that 3 main stress trajectories existed in the facial region, namely the nasomaxillary, zygomaticomaxillary and pterygomaxillary trajectories [7]. The facial functional system was in the mechanical equilibrium between the dentition, muscles and bones [8], the instability of the maxillary segments caused by the maxillary buttress defect could lead to the secondary collapse and displacement of the maxilla [2]. On one hand, the cleft of the UCCLP can destroy the integrity of maxillofacial bone structures and interrupt the physiological occlusal stress transmission; on the other hand, the cleft is located on one side of the midline, the mechanical balance is lost and the stability of the maxillofacial structures will be affected, which in turn has a negative impact on the growth and development of the maxillofacial region. Harikrishnan et al. [9] and Zhao et al. [10] confirmed that the stress distribution between the cleft and non-cleft side of congenital unilateral maxilla cleft was asymmetrical and uneven through FEM (finite element method).

Bone graft in the cleft is the only method for alveolar cleft repairing currently. The most ideal alveolar cleft bone graft is full maxilla cleft bone graft, however, the shape of the cleft is extremely irregular, so it is difficult to achieve this goal, instead, the commonly used secondary full alveolar cleft bone graft is adopted in the clinic since it was reported by Boyne and Sands in 1972 [11]. Yang et al. [12] had found that the stress–strain distribution became more symmetrical during maxilla anterior traction after alveolar cleft bone graft than before. Nagasao et al. [13] had applied uniform loads to the maxilla, alveolar and anterior side of the teeth to simulate upper lip pressure in UCCLP and found that the increased upper lip pressure exacerbated the facial asymmetry, which was alleviated by alveolar cleft bone graft. In summary, alveolar cleft bone graft can restore the integrity of bone segment and provides a support for the canine to erupt (otherwise the patient will lose the teeth), stabilize the bone segment, reconstruct the force conduction, distribute the stress of the maxilla cleft uniformly and alleviate the facial asymmetry.

Meanwhile, bone resorption after alveolar cleft bone graft is a common problem which has puzzled clinicians for a long time, the overall resorption rate was 10.4–100% [14–18]. The final effect of bone resorption after full alveolar cleft bone graft is almost always partial alveolar cleft bone graft, the surviving bones may be distributed in different sites of the alveolar cleft. Chen et al. [19] had studied the effect of maxilla anterior traction on the biomechanics of craniofacial bones of UCCLP after alveolar cleft bone graft and the grafted bone resorption by FEM, and found that the distribution of maxillofacial stresses and deformations was better when maxilla anterior traction was applied after bone graft, it was best in the non-resorbed and it was better in the resorbed when the lower part of the grafted bone than the upper part was lost. What is the effect of bones of different sites in the cleft on the maxillofacial biomechanical distributions of UCCLP under occlusal states? There is no report.

What is the effect of alveolar cleft bone graft on the maxillofacial biomechanical stabilities of UCCLP? From the view of maintaining the stabilities of maxillofacial biomechanics, which sites are the key regions of UCCLP that should be ensured especially when grafting? Which sites should be supplemented with bone graft once bone resorption occurs even if the bones in other sites survive? In order to answer the clinical questions above, the research was carried out.

## Results

**Fig. 1:**
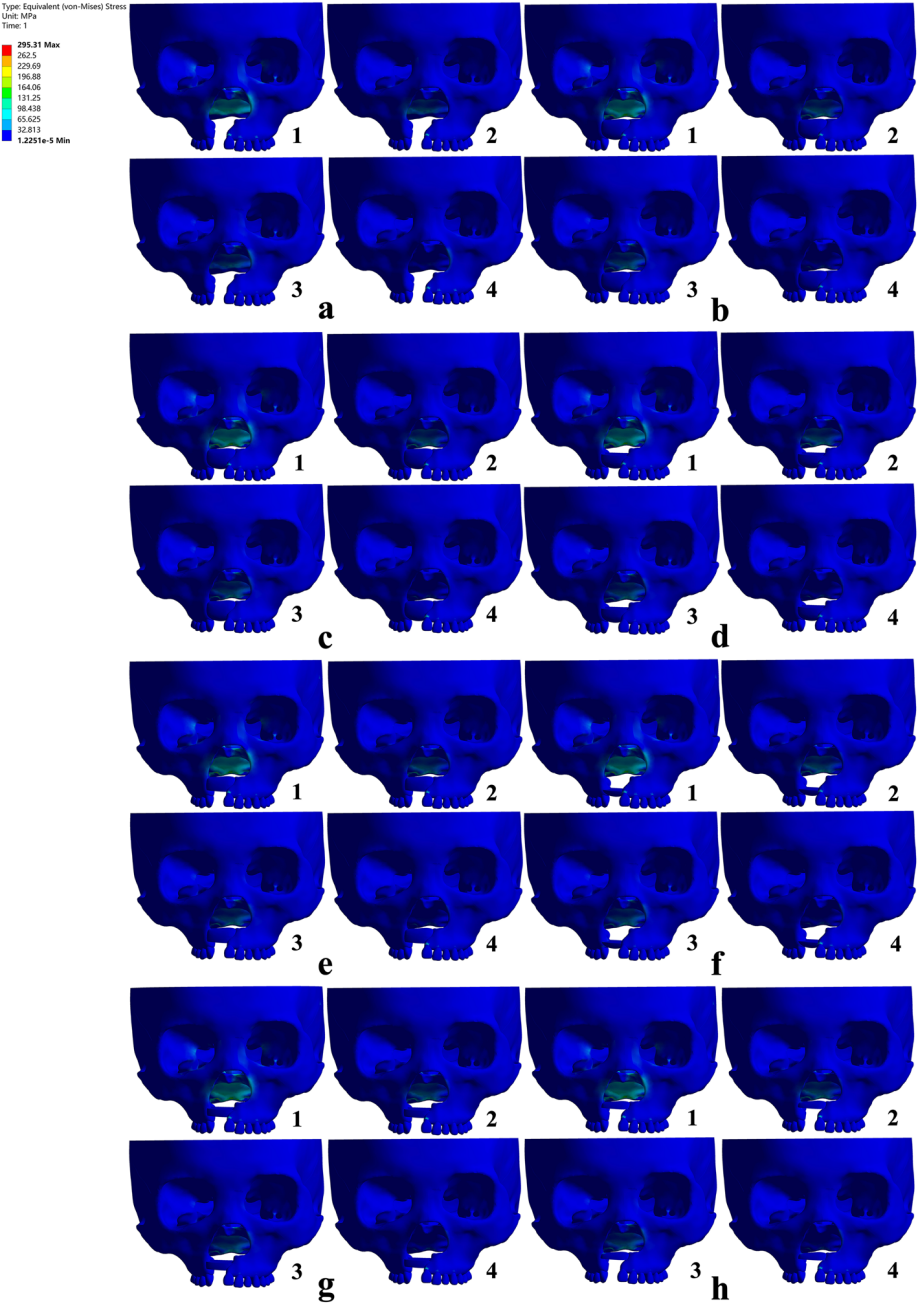
EQV (equivalent) stress nephograms of models under occlusal loads in ANSYS Workbench. The sub-figures were represented, respectively, as: occlusion of the center (**1**), the cleft side (**2**), the non-cleft side (**3**) and the anterior teeth (**4**) on the maxillary dentition of non-bone graft (**a**), full maxilla cleft bone graft (**b**), full alveolar cleft bone graft(**c**), lower 2/3 bone graft (**d**), upper 2/3 bone graft (**e**), lower 1/3 bone graft (**f**), middle 1/3 bone graft (**g**) and upper 1/3 bone graft (**h**) model were simulated

Maxillofacial EQV stress nephograms in non-bone graft model and models with bone graft in different sites of the alveolar cleft of UCCLP under four occlusal states are as in Fig. 1. (Since the overall strain nephograms of maxillofacial bone sutures were too large, they cannot be presented in the article.)

**Fig. 2:**
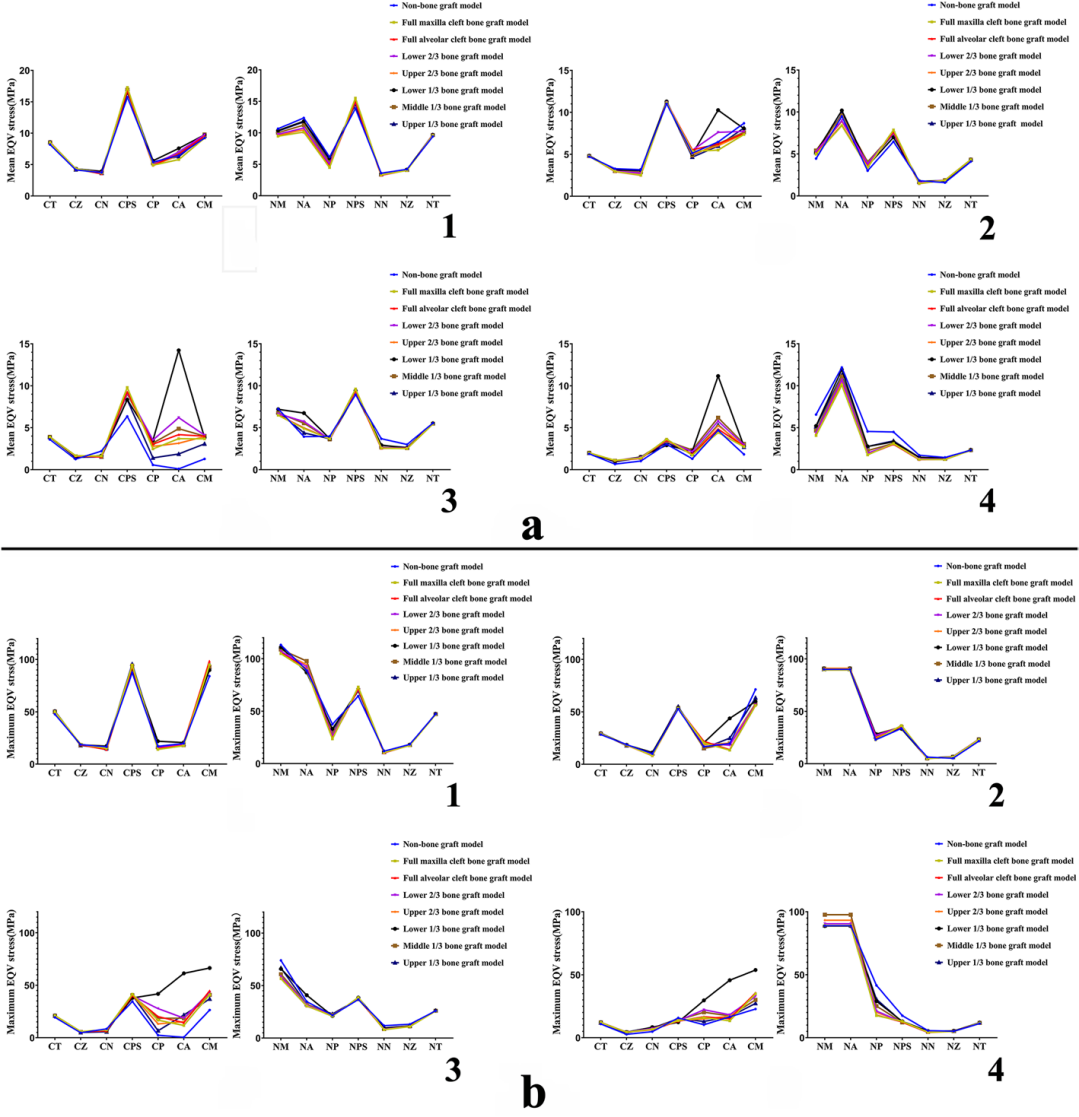
Mean and maximum EQV stress distributions of UCCLP maxillofacial bones. Mean (**a**) and maximum (**b**) EQV stress distributions of maxillofacial bones in non-bone graft model and models with bone graft in different sites of the alveolar cleft under occlusion of the center (**1**), the cleft side (**2**), the non-cleft side (**3**) and the anterior teeth (**4**)

**Fig. 3:**
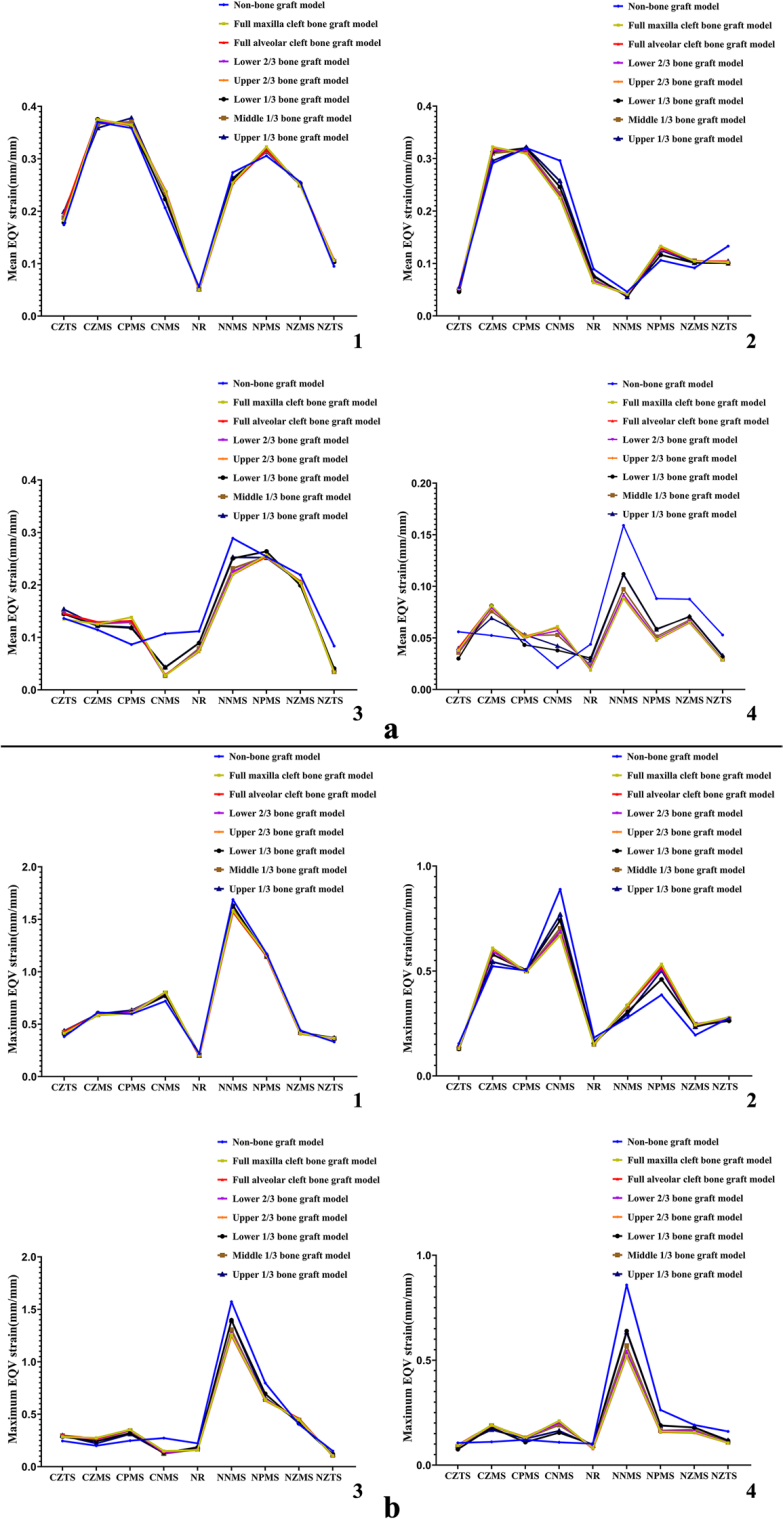
Mean and maximum EQV strain distributions of UCCLP maxillofacial bone sutures. Mean (**a**) and maximum (**b**) EQV strain distributions of maxillofacial bone sutures in non-bone graft model and models with bone graft in different sites of the alveolar cleft under occlusion of the center (**1**), the cleft side (**2**), the non-cleft side (**3**) and the anterior teeth (**4**)

Biomechanical data distributions of UCCLP maxillofacial structures in non-bone graft model and models with bone graft in different sites of the alveolar cleft under four occlusal states are as in Figs. 2, 3.

Three-way ANOVA of biomechanical data distribution variations of UCCLP maxillofacial structures in non-bone graft model and models with bone graft in different sites of the alveolar cleft under four occlusal states are as in Table 1. The type of biomechanical data numbered A, B, C and D represented the mean EQV stresses, maximum EQV stresses, mean EQV strains and maximum EQV strains, respectively, the same as below.

**Table 1:** Three-way ANOVA of the biomechanical data distribution variations of UCCLP maxillofacial structures

Variable (type of biomechanical data)	Square sum of type III	Degree of freedom	Mean square	F	*P*
Models (A)	19.789	7	2.827	9.087	< 0.001
Bones (A)	2865.586	13	220.43	708.587	< 0.001
Occlusal states (A)	1414.802	3	471.601	1515.994	< 0.001
Models * bones (A)	129.153	91	1.419	4.562	< 0.001
Models * occlusal states (A)	15.324	21	0.73	2.346	0.001
Bones * occlusal states (A)	1116.894	39	28.638	92.06	< 0.001
Models (B)	850.863	7	121.552	9.724	< 0.001
Bones (B)	272,022.27	13	20,924.79	1673.977	< 0.001
Occlusal states (B)	41,002.72	3	13,667.573	1093.402	< 0.001
Models * bones (B)	3683.05	91	40.473	3.238	< 0.001
Models * occlusal states (B)	463.89	21	22.09	1.767	0.022
Bones * occlusal states (B)	58,329.084	39	1495.618	119.649	< 0.001
Models (C)	0.003	7	0	3.338	0.002
Bone sutures (C)	0.87	8	0.109	984.689	< 0.001
Occlusal states (C)	1.195	3	0.398	3607.877	< 0.001
Models * bone sutures (C)	0.011	56	0	1.739	0.004
Models * occlusal states (C)	0.003	21	0	1.194	0.262
Bone sutures * occlusal states (C)	1.097	24	0.046	413.78	< 0.001
Models (D)	0.014	7	0.002	1.626	0.131
Bone sutures (D)	16.025	8	2.003	1580.179	< 0.001
Occlusal states (D)	8.855	3	2.952	2328.323	< 0.001
Models * bone sutures (D)	0.118	56	0.002	1.665	0.007
Models * occlusal states (D)	0.017	21	0.001	0.656	0.871
Bone sutures * occlusal states (D)	10.295	24	0.429	338.393	< 0.001

**Fig. 4:**
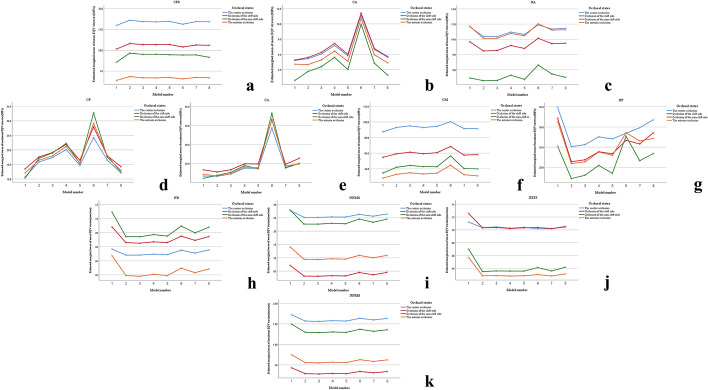
The estimated marginal mean diagrams of statistically significant structural biomechanical data. In different models and under different occlusal states: mean EQV stresses of CPS (**a**), CA (**b**) and NA (**c**); maximum EQV stresses of CP (**d**), CA (**e**), CM (**f**) and NP (**g**); mean EQV strains of NR (**h**), NNMS (**i**) and NZTS (**j**); maximum EQV strains of NNMS (**k**)

Simple effect analysis of biomechanical data distribution variations of the same UCCLP maxillofacial structure in different models under four occlusal states are as in Table 2 and Fig. 4. Since the original tables were too long, only data with statistical significance, i.e., *P* < 0.05 are presented in the article. The model numbered 1 and 2, 3, 4, 5, 6, 7, 8 represents non-bone graft model and full maxilla cleft, full alveolar cleft, lower 2/3, upper 2/3, lower 1/3, middle 1/3, upper 1/3 bone graft model, respectively.

**Table 2:** Simple effect analysis of biomechanical data distribution variations of the same structure

Structure (type of biomechanical data)		Square sum	Degree of freedom	Mean square	F	*P*
CPS (A)	Contrast	5.197	7	0.742	2.387	0.022
	Error	84.926	273	0.311		
CA (A)	Contrast	117.89	7	16.841	54.138	< 0.001
	Error	84.926	273	0.311		
NA (A)	Contrast	10.474	7	1.496	4.81	< 0.001
	Error	84.926	273	0.311		
CP (B)	Contrast	785.5	7	112.214	8.977	< 0.001
	Error	3412.513	273	12.5		
CA (B)	Contrast	2492.739	7	356.106	28.488	< 0.001
	Error	3412.513	273	12.5		
CM (B)	Contrast	613.269	7	87.61	7.009	< 0.001
	Error	3412.513	273	12.5		
NP (B)	Contrast	324.788	7	46.398	3.712	0.001
	Error	3412.513	273	12.5		
NR (C)	Contrast	0.002	7	0	2.419	0.022
	Error	0.019	168	0		
NNMS (C)	Contrast	0.006	7	0.001	7.201	< 0.001
	Error	0.019	168	0		
NZTS (C)	Contrast	0.002	7	0	2.421	0.022
	Error	0.019	168	0		
NNMS (D)	Contrast	0.102	7	0.015	11.46	< 0.001
	Error	0.213	168	0.001

Table 2 and Fig. 4a–c show that the mean EQV stresses of CPS, CA and NA are significantly different in different models (*P* < 0.05). The mean EQV stresses of CPS are significantly higher in model 2 than in model 1 (*P* < 0.05), the mean EQV stresses of CA are significantly higher in model 6 than in other models (*P* < 0.05), the mean EQV stresses of CA are significantly higher in model 4 than in model 1, 2, 5 and 8 (*P* < 0.05), the mean EQV stresses of CA are significantly higher in model 7 than in model 1 (*P* < 0.05), the mean EQV stresses of NA are significantly higher in model 6 than in model 2, 3 and 5 (*P* < 0.05).

Table 2 and Fig. 4d–g show that the maximum EQV stresses of CP, CA, CM and NP are significantly different in different models (*P* ≤ 0.001). The maximum EQV stresses of CP are significantly higher in model 6 than in model 1, 2, 3, 5, 7 and 8 (*P* < 0.05), the maximum EQV stresses of CA are significantly higher in model 6 than in other models (*P* < 0.05), the maximum EQV stresses of CM are significantly higher in model 6 than in other models (*P* < 0.05), the maximum EQV stresses of NP are significantly higher in model 1 than in model 2, 3 and 5(*P* < 0.05).

Table 2 and Fig. 4h–j show that the mean EQV strains of NR, NNMS and NZTS are significantly different in different models (*P* < 0.05). The mean EQV strains of NR are significantly higher in model 1 than in model 3 (*P* < 0.05), the mean EQV strains of NNMS are significantly higher in model 1 than in other models (*P* < 0.05), the mean EQV strains of NZTS are significantly higher in model 1 than in model 4 and 7 (*P* < 0.05).

Table 2 and Fig. 4k show that the maximum EQV strains of NNMS are significantly different in different models (*P* < 0.001). The maximum EQV strains of NNMS are significantly higher in model 1 than in other models (*P* < 0.001).

### Summary of the results

The main purpose of the research is to explore the effect of alveolar cleft bone graft on UCCLP maxillofacial biomechanics, which sites should be supplemented with bone graft once bone resorption occurs. The most ideal alveolar cleft bone graft method—full maxilla cleft bone graft is difficult to achieve, so the commonly used full alveolar cleft bone graft is adopted. Therefore, the main comparative approach in the research was to use the biomechanical data of full alveolar cleft bone model as the standard, the biomechanical data of maxillofacial structures of models with bone graft in different sites of the alveolar cleft were compared with the standard.

The EQV stress distributions of UCCLP maxillofacial bones in different models under four occlusal states and the statistical analysis are shown as follows.

The mean and maximum EQV stresses of anterior alveolar arches on the non-cleft side, bilateral maxillae and pterygoid processes of sphenoid bones were all higher than other maxillofacial bones under the centric occlusion. The mean EQV stresses of pterygoid processes of sphenoid bones on the cleft side were all higher than other maxillofacial bones, the maximum EQV stresses of anterior alveolar arches on the non-cleft side, maxillae and pterygoid processes of sphenoid bones on the cleft side were all higher than other maxillofacial bones under occlusion of the cleft side. The maximum EQV stresses of anterior alveolar arches on the non-cleft side were all higher than other maxillofacial bones under the anterior occlusion.

The maximum EQV stresses of posterior alveolar arch on the non-cleft side of full alveolar cleft bone graft model were significantly lower than non-bone graft model. The mean EQV stresses of bilateral anterior alveolar arches of lower 1/3 bone graft model were significantly higher than full alveolar cleft bone graft model, the maximum EQV stresses of maxilla and its alveolar arch on the cleft side of lower 1/3 bone graft model were significantly higher than full alveolar cleft bone graft model. There was no significant statistical difference in the EQV stress distributions of maxillofacial bone structures between full maxilla and full alveolar cleft bone graft model.

There was no significant difference in the EQV stress distributions of bilateral nasal bones, zygomata, temporal bones and maxillae, pterygoid processes of sphenoid bones on the non-cleft side of all models under four occlusal states. The EQV stresses of bilateral nasal bones and zygomata were generally lower than other maxillofacial bones.

The EQV strain distributions of UCCLP maxillofacial bone sutures in different models under four occlusal states and the statistical analysis are shown as follows.

The mean EQV strains of bilateral nasomaxillary, pterygomaxillary and zygomaticomaxillary sutures were all higher than other maxillofacial bone sutures, the maximum EQV strains of bilateral nasomaxillary sutures, pterygomaxillary sutures on the non-cleft side were all higher than other maxillofacial bone sutures under the centric occlusion. The mean and maximum EQV strains of ipsilateral nasomaxillary, pterygomaxillary and zygomaticomaxillary sutures were all higher than other maxillofacial bone sutures under occlusion of the cleft or the non-cleft side. The mean and maximum EQV strains of nasomaxillary sutures on the non-cleft side were all higher than other maxillofacial sutures under the anterior occlusion.

The mean EQV strains of nasal raphe and nasomaxillary suture on the non-cleft side of full alveolar cleft bone graft model were significantly lower than non-bone graft model, and the maximum EQV strains of nasomaxillary suture on the non-cleft side of full alveolar cleft bone graft model were significantly lower than non-bone graft model. There was no significant statistical difference in the EQV strain distributions between models with bone graft in other sites of the alveolar cleft and full alveolar cleft bone graft model.

There was no significant difference in the EQV strain distributions of bilateral pterygomaxillary, zygomaticomaxillary sutures and nasomaxillary, zygomaticotemporal sutures on the cleft side of all models under four occlusal states.

## Discussion

Approximately 75% of patients with cleft lip and palate have varying degrees of alveolar cleft [20]. UCLP (Unilateral Cleft Lip and Palate) deformity has a significant impact on the bone morphology of the mid-facial and oronasal regions [21], the alveolar process on the non-cleft side is more prominent and protuberant than that on the cleft side [2]. There are asymmetrical bone movements in UCLP due to the special maxillofacial structure [22], loss of bone and soft tissue on the cleft side and tension of the repaired lip on the non-cleft side can lead to flattening and recession of the central face [23]. Alveolar cleft bone graft is a key step in the sequential treatment for cleft lip/palate [24], a prominent function of it is stabilizing the maxillary dental arch [25], prevent re-collapse of the expanded maxillary segments [26]. Studies have shown that one reason for bone resorption after alveolar cleft bone graft was the lack of proper physiological stress stimulation [17, 27–29].

In order to promote permanent teeth eruption near the cleft, bone connectivity and adequate bone thickness are necessary [30]. Since it is believed that primary (2–5 years) alveolar cleft bone graft can inhibit the growth and development of maxilla (although it is not fully confirmed), secondary alveolar cleft bone graft is widely used at present, it can be divided into early secondary bone graft (about 7–11 years) and late secondary bone graft (about 14–18 years) [31]. The research results of different medical institutions also showed a minor difference in the optimal time of alveolar cleft bone graft, mainly: 6–9 [32], 8–12 [33], 9–11 [31] and 9–12 [34] years. It is closely related to the development of permanent maxillary canine roots [31], i.e., after eruption of permanent incisors and before eruption of permanent canines [35], alveolar cleft bone graft is usually performed when the development of the adjacent unerupted permanent canine roots is 1/4 to 2/3 complete [36]. However, late alveolar cleft bone graft can still acquire acceptable surgical effects in some situations although the optimal age for the surgery is missed [37]. The case elected in the research was an adult typical UCCLP patient, the reason is that the grafted materials in the cleft are usually unstable in the short term, however, the aim of the research was to explore the long-term effect of alveolar cleft bone graft on the biomechanical stabilities of UCCLP under occlusal states after total fusion and ossification of the entire or partial grafted materials with the cleft ends, so the biomechanical parameters of the grafted materials were also set according to the normal cancellous bone [38].

The accuracy of the results mainly depends on the accuracy of the modeling process in finite element researches [39]. The experiment integrated previous research outcomes and periodontal membranes with 0.2 mm thicknesses [40–47] were reconstructed on the root surfaces. In order to conform to the clinical reality as much as possible, the experiment was performed by dividing maxillofacial bones with reference to the original bone sutures, 0.2 mm widths [48–50] were adopted on the bone sutures when the gaps between maxillofacial bones were measured in Siemens NX meanwhile according to the age of the patient elected. The inherent properties of bones [51–54] and periodontal membranes [55–59] are anisotropic, bones [60] and periodontal membranes [46] are biphasic materials consisting of solid and liquid phases, meanwhile, bones [61–63], bone sutures [64, 65] and periodontal membranes [66] have viscoelastic properties. Since bone does not exhibit a large number of time-dependent material behaviors generally, the viscoelastic effect of the bone liquid phase can be neglected [67–69]. Periodontal membrane deforms in a viscoelastic pattern when subjected to small continuous forces [70], however, because the fluid does not have enough time to flow, the periodontal membrane responds elastically and linearly to an instantaneous [71], large load (mastication) [72]. Therefore, it is sufficient to assume that periodontal ligaments are isotropic and elastic when teeth are displaced instantaneously [73]. The hard tissue of teeth is very rigid that behaves like rigid bodies [74] and the measurement results of tooth movements found in the cadaveric materials were highly linear [55]. Viscoelastic properties of skull models are essential in the analysis involving lower frequencies, however, they are not necessary in simulating transient loads such as chewing [65]. Kabel et al. [75] had demonstrated that the apparent elastic properties could be estimated by using isotropic and homogeneous tissue moduli. Therefore, the properties of structures in models were set to be linear, elastic and isotropic.

The research was conducted under rather larger occlusal loads [2, 6] with the aim of highlighting the supporting roles of bony buttresses in the maxillofacial region. Gross et al. [76] found the strains increased on the alveolar arch and the nasal margin when simulated occlusal loads applied to the entire maxillary dental arch. Alexandridis et al. [7] found that stresses generated by occlusal loads were transmitted through maxilla along the nasal, zygomatic and pterygoid process pathways, in the zygomatic region, stresses were distributed posteriorly along the zygomatic arch to the temporal bone. Alexandridisi et al. [8] also found that in the midface, especially in the zygomatic region, the main effect of masseters on the zygomatic–temporal trajectory was very pronounced and most of the maxillary loads were borne by the region. It was also confirmed that the main stress trajectory of zygoma and zygomatic arch followed alveolar–maxillary–zygomatic–temporal bone direction, and the stresses were highly concentrated in the zygomatic process of temporal bone [8]. The lateral maxilla was found to be the main vertical buttress in normal maxilla under maximum occlusal force by Pakdel et al. [77], the nasomaxillary buttress bore less loads, however there were insufficient evidences showed that the pterygomaxillary region to be a buttress structure. For UCCLP maxillofacial biomechanics, Pan et al. [78] found significant asymmetric displacements and deformations of UCLP under the effects of maxillary expansion by FEM. Zhao et al. [10] initially analyzed the stress–strain distribution in the maxillary alveolar region of UCCLP under typical functional loads, it showed that the palatal deformity resulted in asymmetric stress and strain distributions with higher stress and strain levels on the non-cleft side. Zhao et al. [79] also found that unilateral maxilla cleft resulted in uneven and asymmetric stress–strain distribution within the maxilla with strengthening on the non-cleft side while weakening on the cleft side. Gautam et al. [80] had evaluated the effects of maxillary expansion on the skeleton of UCLP by FEM and found that the regions with the maximum stress values were the primary palatal region, the infraorbital foramina on both sides and the zygomatic buttress on the cleft side. Lee et al. [81] had predicted the optimal force application points for UCLP expansion and found that high stress concentrations were observed in the pterygoid body, medial orbital and lower maxillary zygomatic processes, higher stress levels were observed on the cleft side, the stresses were also distributed along the trajectories of nasomaxillary, zygomaticomaxillary and pterygomaxillary buttresses when forces were applied on the maxillary teeth. Harikrishnan et al. [9] found that the normal cranium exhibited significant nasomaxillary, zygomaticomaxillary and pterygomaxillary buttress mechanic transfer trajectory actions under bilateral posterior occlusal loads by FEM. Whereas the role of nasomaxillary buttress was more pronounced on the cleft side than on the non-cleft side, the role of zygomaticomaxillary and pterygomaxillary buttress was more pronounced on the non-cleft side than on the cleft side in unilateral maxilla cleft under the same loads [9]. However, Yang et al. [12] and Nagasao et al. [13] found that alveolar cleft bone graft could alleviate the asymmetry of stress–strain distributions in UCCLP.

The results were in accordance with the conclusions of Gross et al. [76] and Pakdel et al. [77], it is evident that bilateral maxillae and their alveolar arches still play the central roles for occlusal load-bearing in UCCLP. The results showed that nasomaxillary and zygomaticomaxillary buttress of UCCLP did not play much strong occlusal load-bearing roles, whereas the role of the pterygomaxillary buttress was obvious, which significantly differed from the findings of Gross et al. [76] and Alexandridisi et al. [8], however, there were both similarities and differences with the findings of Pakdel et al. [77], Gautam et al. [80], Lee et al. [81] and Harikrishnan et al. [9]. The reason may be that pterygoid processes of sphenoid bones of UCCLP share more occlusal loads, thus the load-bearing roles of nasal bones and zygomata become weaken. From the data statistical analysis, it can be concluded that the area that be affected by alveolar cleft bone graft on the occlusal stress of UCCLP maxillofacial bones is mainly in the posterior alveolar arch on the non-cleft side. The results are also consistent with Chen et al. [19], and demonstrated that: bone resorption near nasal base and in the middle of the alveolar cleft can significantly increase occlusal loads borne by bilateral anterior alveolar arches, it can also significantly enhance the concentration of occlusal stresses in the maxilla and its alveolar arch on the cleft side. However, the effect of the rest grafted bone resorption in the alveolar cleft on the biomechanics of maxillofacial bones under occlusal loads is not significant. It has been shown that bone resorption after alveolar cleft bone graft was also mostly observed in the root and palatal parts of the cleft [82], so the research results are consistent with common clinical realities on some degree.

Alexandridis et al. [7] found that occlusal stresses generated from closed-mouth muscles by mandible were concentrated on nasofrontal, zygomaticomaxillary and pterygopalatal suture, and in zygomatic region stresses were distributed upward to zygomaticofrontal suture and backward along the zygomatic arch to zygomaticotemporal suture. It is known from the results that nasomaxillary, pterygomaxillary and zygomaticomaxillary sutures of UCCLP are the main bone sutures bearing occlusal loads. It also confirms that occlusal loads can be transmitted along nasomaxillary, zygomaticomaxillary and pterygomaxillary buttresses through corresponding bone sutures. Alveolar cleft bone graft can significantly reduce occlusal loads borne by nasal raphe and nasomaxillary suture on the non-cleft side, and also significantly weaken the concentration of occlusal strains on nasomaxillary suture on the non-cleft side in UCCLP. It is demonstrated that alveolar cleft bone graft mainly affects the strain distribution of bone sutures above under occlusal loads. There are no significant differences in EQV strain distributions on bilateral pterygomaxillary, zygomaticomaxillary sutures and nasomaxillary, zygomaticotemporal suture on the cleft side before and after alveolar cleft bone graft, it is known that strains of the bone sutures above under corresponding occlusal loads are not affected by presence or absence of the grafted bone in the alveolar cleft.

Deficiencies of the study and further research directions: (1) maxilla clefts contain many types, the effects of alveolar cleft bone graft on maxillofacial biomechanics of other maxilla cleft types should be carried out in the future to provide clinical guidance for bone graft in other types of maxilla clefts. (2) The purpose of this research was to explore the effects of alveolar cleft bone graft on maxillofacial biomechanics under occlusal states after the grafted bone was complete fusion with both ends of the clefts and complete ossification. The effects of different ossification stages of the grafted bone in the alveolar cleft on the maxillofacial biomechanics under occlusal states can be researched in the future to explore the effects of alveolar cleft bone graft on maxillofacial biomechanics in more detail.

## Conclusions

For UCCLP:

1. Bilateral maxillae, pterygoid processes of sphenoid bones and bilateral nasomaxillary, zygomaticomaxillary, pterygomaxillary sutures, anterior alveolar arch on the non-cleft side are the main bearing structures for occlusal loads before and after alveolar cleft bone graft.
2. Alveolar cleft bone graft mainly affects biomechanical stabilities of nasal raphe and posterior alveolar arch, nasomaxillary suture on the non-cleft side under occlusal loads.
3. The areas near nasal floor and in the middle of the alveolar cleft are the sites that needed to be guaranteed when bone grafting, and supplementary bone graft should be performed when the grafted bone in these areas of the alveolar cleft resorbed.

## Methods

### Equipment and software

CT data acquisition equipment: Philips MX 16-slice X-ray electron computed tomography device (Philips Electronics, Netherlands).

Software: Mimics 20 (Materialise, Belgium), Geomagic Studio 2014 (3D Systems, USA), Siemens PLM NX 12.0.0 (Siemens, Germany), ANSYS Workbench 19.2 (ANSYS, USA).

### Materials

A 24-year-old female typical UCCLP patient without skeletal systemic disorders, severe dropout of bone segments on both sides of the cleft and severe dentofacial plane deviation, also not undergoing maxillary orthopedic and orthodontics was selected. The patient underwent cheiloplasty at the age of 4, cleft palate repair and nasolabial deformity revision at the age of 23. The patient's skull and neck were scanned by Philips MX 16-slice X-ray electron computed tomography device before alveolar cleft bone graft, the DICOM (Digital Imaging and Communications in Medicine) format data of CT were obtained. The use of the patient's CT data for the research was conducted with the patient's consent and approved by Medical Ethics Committee of West China Hospital of Stomatology, Sichuan University (Grant No. WCHSIRB-D-2020-362).

### Establishment of UCCLP maxillofacial 3D CAD models of non-bone graft and bone graft in different sites of the alveolar cleft

**Fig. 5:**
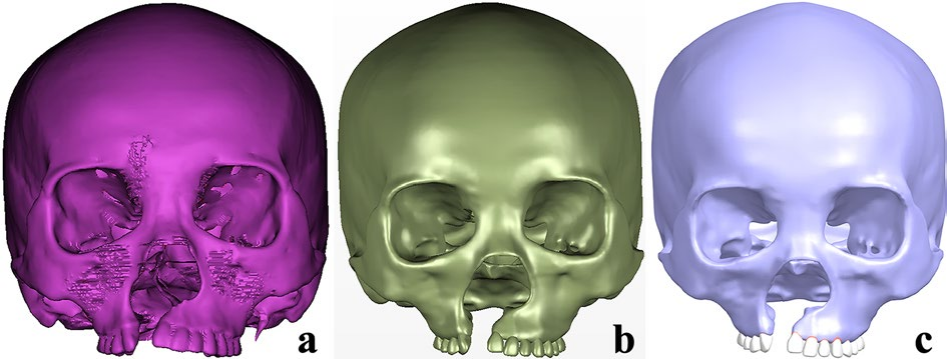
UCCLP craniomaxillofacial 3D CAD model: **a** the structural images not much relevant to the research were erased in Mimics, the model in.stl was obtained; **b** the model in.step was obtained by further repairing, smoothing, finely modeling of planes and curved surfaces in Geomagic Studio; **c** assembly of the maxillary teeth, the periodontal ligaments and the craniomaxillofacial bone in Siemens NX, the model in.prt was obtained

The DICOM data were imported into Mimics, the cervical spines, hyoid bone and mandible with its dental images were removed, the model in.stl (StereoLithography) was generated as shown in Fig. 5a, the microdontia was removed, the small holes were repaired, the surfaces of bones and teeth were trimmed. The model was then imported into Geomagic Studio, and further repaired, smoothed, finely modeled in planes and curved surfaces to obtain the model in.step (standard for the exchange of product model data), as shown in Fig. 5b, the root surfaces of the maxillary teeth were expanded outward by 0.2 mm [40–47], the original alveolar sockets of the teeth were fitted to generate the periodontal ligaments of the tooth root surfaces by Boolean operations. The maxillary teeth, the periodontal ligaments and the craniomaxillofacial bone were imported into Siemens NX for assembly to obtain the 3D (three-dimensional) CAD (computer aided design) model in.prt, as shown in Fig. 5c.

**Fig. 6:**
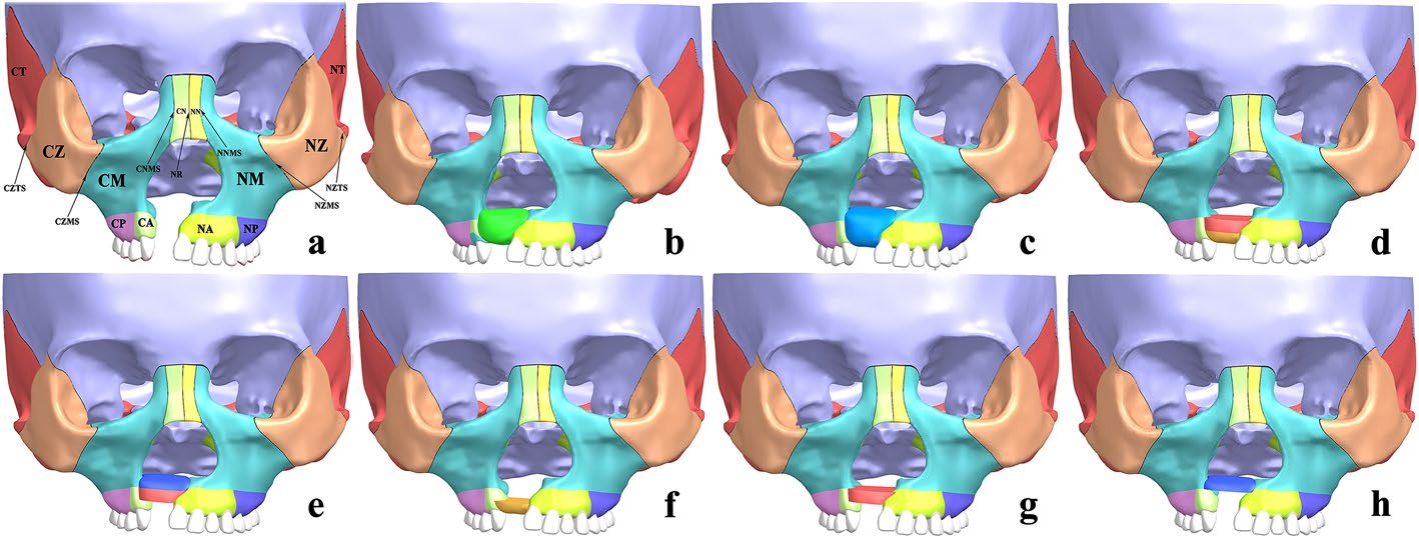
UCCLP maxillofacial 3D CAD models of non-bone graft and alveolar cleft bone graft. Maxillofacial 3D CAD models of non-bone graft (**a**), full maxilla cleft (**b**) and full alveolar cleft (**c**) bone graft, bone graft in lower 2/3 **(d**), upper 2/3 (**e**), lower 1/3 (**f**), middle 1/3 (**g**) and upper 1/3 (**h**) of the alveolar cleft according to the height with nasal floor as the upper surface and the alveolar ridge crest side as the lower surface

With the reference to bone sutures in the 3D CT reconstructed maxillofacial image, bones were depicted and segmented: CM (maxilla on the cleft side), CA (anterior alveolar arch on the cleft side), CP (posterior alveolar arch on the cleft side), CPS (pterygoid process of sphenoid bone on the cleft side), CN (nasal bone on the cleft side), CZ (zygoma on the cleft side), CT (temporal bone on the cleft side) and NM (maxilla on the non-cleft side), NA (anterior alveolar arch on the non-cleft side), NP (posterior alveolar arch on the non-cleft side), NPS (pterygoid process of sphenoid bone on the non-cleft side), NN (nasal bone on the non-cleft side), NZ (zygoma on the non-cleft side), NT (temporal bone on the non-cleft side). Bone sutures with 0.2 mm widths [48–50] were depicted and reconstructed at the junctions of adjacent bones, the research mainly focused on bone sutures with maxilla buttress as the center: CNMS (nasomaxillary suture on the cleft side), CPMS (pterygomaxillary suture on the cleft side), CZMS (zygomaticomaxillary suture on the cleft side), CZTS (zygomaticotemporal suture on the cleft side), NR(nasal raphe), NNMS (nasomaxillary suture on the non-cleft side), NPMS (pterygomaxillary suture on the non-cleft side), NZMS (zygomaticomaxillary suture on the non-cleft side), NZTS (zygomaticotemporal suture on the non-cleft side).The cranial bones above the top of bilateral temporal bones were removed to obtain UCCLP maxillofacial CAD model in.prt, hereafter referred to as non-bone graft model, as shown in Fig. 6a.

Non-bone graft model was imported into Siemens NX to generate models of bone graft within full maxilla cleft (hereinafter referred to as full maxilla cleft bone graft model, as shown in Fig. 6b) and full alveolar cleft bone graft model (hereinafter referred to as full alveolar cleft bone graft model, as shown in Fig. 6c), respectively. The 3D CAD model of the grafted bone in the full alveolar cleft was divided into three equal parts according to the height with the nasal floor side as the upper surface and the alveolar ridge crest side as the lower surface. Comprehensively considering the possible bone resorption situations at different heights of the grafted bone after full alveolar cleft bone graft in the clinic, the middle 1/3 + lower 1/3, upper 1/3 + middle 1/3, lower 1/3, middle 1/3 and upper 1/3 part of the grafted bone in the full alveolar cleft were assembled with the original junctions at two ends of the cleft, respectively, to form 5 models to simulate the rest grafted bone resorption, as shown in Fig. 6d–h. The 5 models were hereafter referred to as lower 2/3, upper 2/3, lower 1/3, middle 1/3 and upper 1/3 bone graft model, respectively, together with non-bone graft model, full maxilla cleft and full alveolar cleft bone graft model, a total of 8 UCCLP maxillofacial CAD models of non-bone graft and bone graft in different sites of the alveolar cleft were formed.

### Different occlusal loads on UCCLP maxillofacial regions of models

The occlusal plane was formed by the mesial contact point of the maxillary central incisors and the mesial buccal cusp apexes of bilateral first maxillary molars in Siemens NX [83]. Models were imported into ANSYS Workbench, the properties of all structures were set to be linear, elastic and isotropic, Young’s moduli and Poisson’s ratios of structures were set according to Table 3 [38, 84, 85], the contact relationships of all adjacent structures were set to “bonded” (no sliding or separation between faces or edges is allowed). According to the general rules of FEM using, mesh convergence test was performed on the model [86], the tetrahedral mesh dimensions of bones, periodontal ligaments and bone sutures, teeth of the non-bone graft model were assumed in descending order as follows: 5, 1, 2 mm, 4, 0.75, 1.5 mm, 3, 0.5, 1 mm and 2, 0.25, 0.5 mm, the maximum EQV stress (MPa) and the maximum displacement (mm) of the entire model under the simulated centric occlusion according to Table 4 [2, 6] were judged as the indexes, it was found that the most suitable convergence effect was obtained when the dimensions of 3, 0.5, 1 mm were adopted for tetrahedral meshes of bones, periodontal ligaments and bone sutures, teeth of the model, respectively, so structures of all imported models were tetrahedrally meshed according to the most suitable dimensions above. Tetrahedral meshing results of models are shown in Additional file 1: Fig. S1, sub-figs of Additional file 1: Fig. S1 represent, respectively, as: tetrahedral meshing results of non-bone graft model (a) and full maxilla cleft (b), full alveolar cleft (c), lower 2/3 (d), upper 2/3 (e), lower 1/3 (f), middle 1/3 (g), upper 1/3 (h) bone graft model, the numbers of elements and nodes obtained by tetrahedral meshing of models are shown in Additional file 3: Table S1. The occipital foramen magnum was set as the fixed constraint [87], the forces were loaded on the thrust surfaces of corresponding teeth, the directions of the loaded forces were perpendicular to the occlusal plane, the force values were set as in Table 4 [2, 6], occlusal load diagrams are shown in Additional file 2: Fig. S2, sub-figs of Additional file 2: Fig. S2 represent, respectively, as: occlusal load diagrams of the center (1), the cleft side (2), the non-cleft side (3) and the anterior teeth (4) on maxillary dentition of non-bone graft model(a) and full maxilla cleft (b),full alveolar cleft (c), lower 2/3 (d), upper 2/3 (e), lower 1/3 (f), middle 1/3 (g), upper 1/3 (h) bone graft model, respectively.

**Table 3:** Young's moduli and Poisson's ratios of structures

Structures	Young's modulus(MPa)	Poisson’s ratio
Craniomaxillofacial bones	13,700	0.3
Grafted bone in the cleft	7900	0.3
Teeth	20,000	0.3
Periodontal ligaments	0.49	0.49
Bone sutures	7	0.4

**Table 4:** Forces loaded on the maxillary dentition under four occlusal states

Tooth area	The cleft side			The non-cleft side		
Force value(N)						
Occlusal state	Anterior teeth area	Premolar area	Molar area	Anterior teeth area	Premolar area	Molar area
The centric occlusion	160	280	400	160	280	400
Occlusion of the cleft side	160	280	400	/	/	/
Occlusion of the non-cleft side	/	/	/	160	280	400
The anterior occlusion	160	/	/	160	/	/

### Analysis indexes

#### Maxillofacial bones

EQV stress: also known as von Mises stress. When an object is subjected to an external force, an internal force is generated within the object that resists the external force and restores the object from its post-deformation position to its pre-deformation position, the internal force per unit area at a point in its cross section is stress. von Mises stress reflects the stress state inside a structure by the stress contour, which can depict the stress variations in the structure after a load is applied. EQV stress is widely used in biomechanical researches [87] and one of the gold standards for evaluating bone stress distributions [88], therefore, EQV stresses were adopted as the indexes for analysis of maxillofacial bone biomechanical variations.

#### Maxillofacial bone sutures

EQV strain: the deformation per unit length of an object under stress is strain. The total strain component is calculated by applying various types of loads to the object, then EQV strain is calculated from the total deformation component. Since bone sutures have a certain degree of movability [39], so EQV strains were used as the analysis indexes of biomechanical variations in maxillofacial bone sutures. The EQV strain type used in the research was elastic strain.

The mean EQV stress or strain is the mean value of EQV stresses or strains of the whole structure, which indicates the whole EQV stress or strain state of the structure; while the maximum EQV stress or strain is the maximum value of the EQV stresses or strains of the structure, which is located in a point on the structure and reflects the stress or strain concentration trend of the structure.

### The statistical method

Three-way ANOVA was used to analyze the biomechanical data distribution variations of UCCLP maxillofacial structures with *P* < 0.05 as the statistical difference.

## Supplementary Information

**Additional file 1:** **Fig. S1** Tetrahedral meshing results of models.

**Additional file 2:** **Fig. S2** Occlusal load diagrams.

**Additional file 3:** **Tab. S1** The numbers of elements and nodes obtained by tetrahedral meshing of models.

## Data Availability

All the data and materials of the research are available.

## Abbreviations

UCCLP: Unilateral complete cleft lip and palate
FEM: Finite element method
EQV: Equivalent
UCLP: Unilateral cleft lip and palate
DICOM: Digital Imaging and Communications in Medicine
.stl: StereoLithography
.step: Standard for the exchange of product model data
3D: Three-dimensional
CAD: Computer aided design
