# Supplementary figures and images for: Three-dimensional finite element analysis of the effect of alveolar cleft bone graft on the maxillofacial biomechanical stabilities of unilateral complete cleft lip and palate
Source: Biomed Eng Online. 2022 May 20;21:31. doi: 10.1186/s12938-022-01000-y (PMC9123812; doi:10.1186/s12938-022-01000-y)

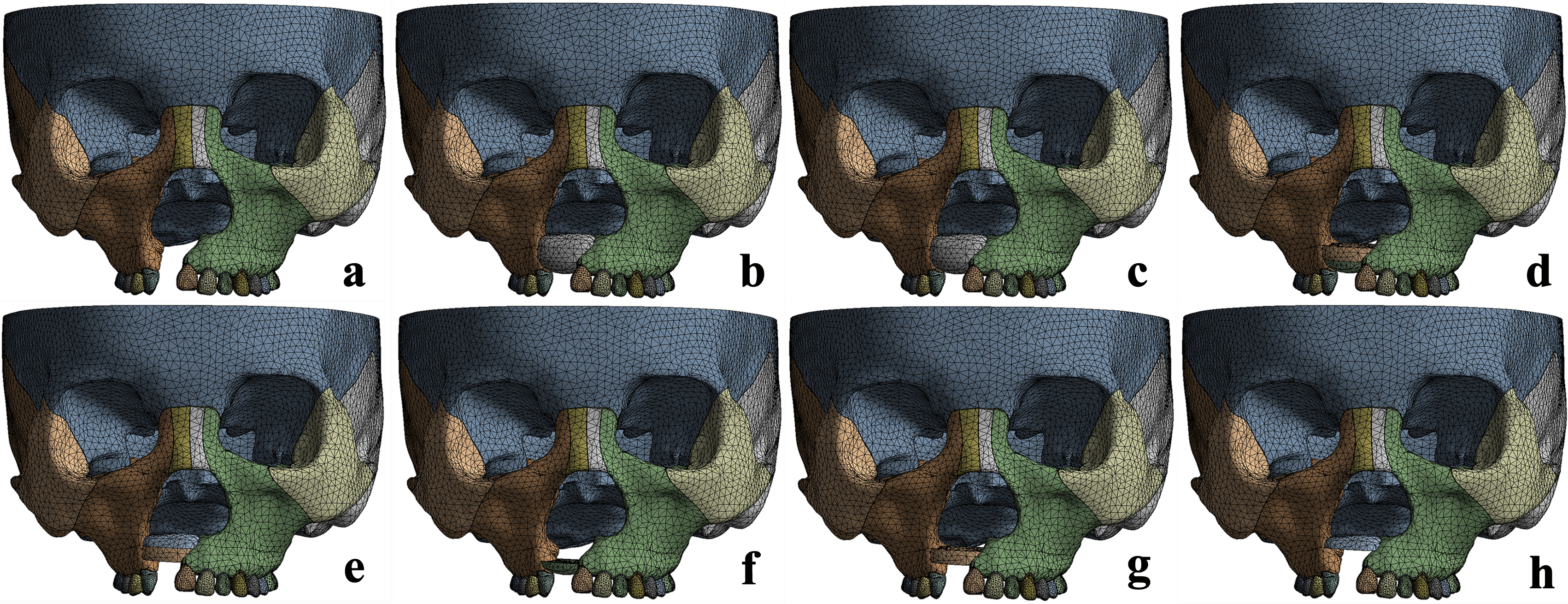

Supplement: Supplementary file 1 — Additional file 1: Fig. S1 Tetrahedral meshing results of models. [file 12938_2022_1000_MOESM1_ESM.png]

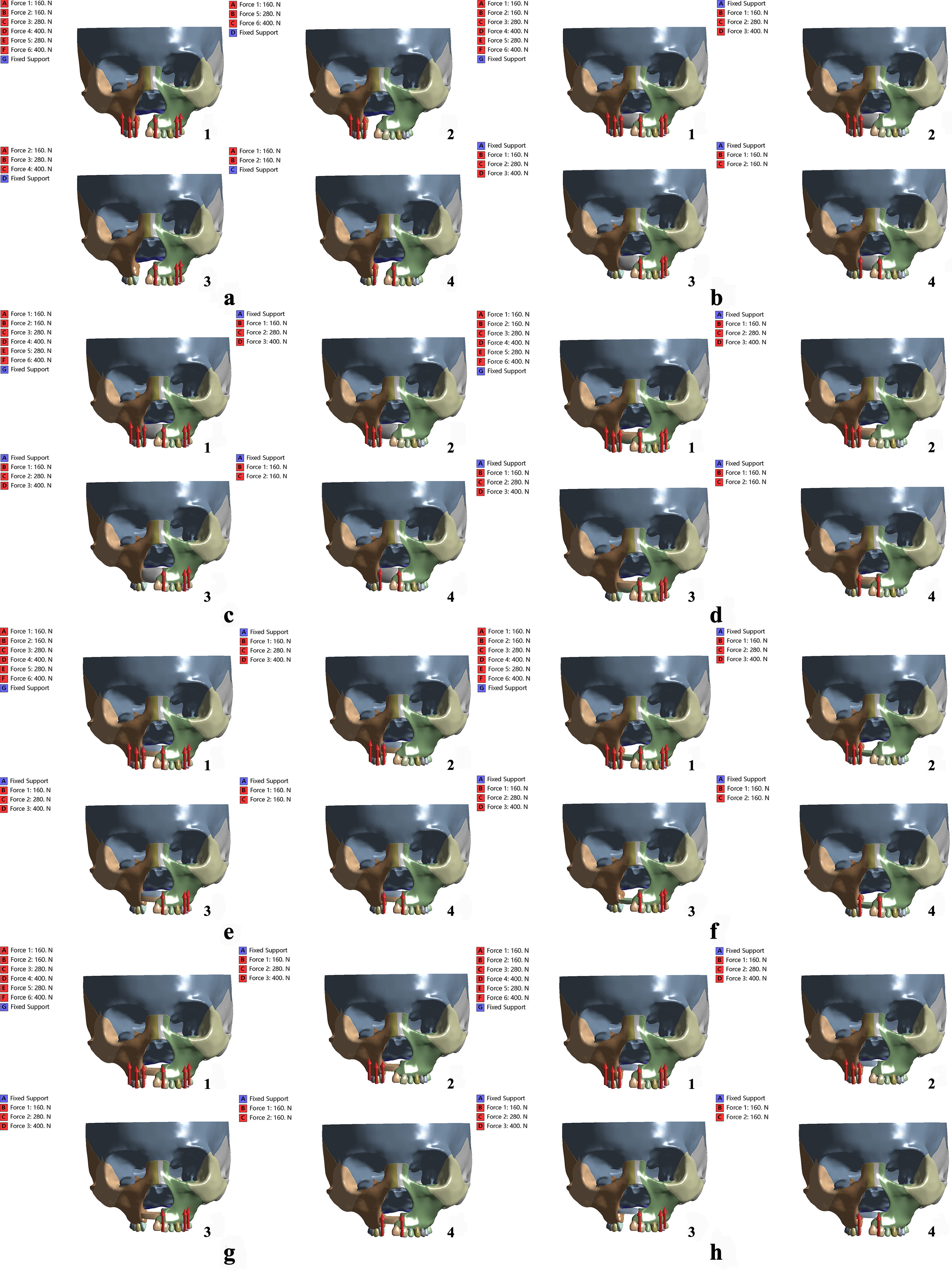

Supplement: Supplementary file 2 — Additional file 2: Fig. S2 Occlusal load diagrams. [file 12938_2022_1000_MOESM2_ESM.png]
