# Supplementary material for: Three-dimensional finite element analysis of the effect of alveolar cleft bone graft on the maxillofacial biomechanical stabilities of unilateral complete cleft lip and palate
Source: Biomed Eng Online. 2022 May 20;21:31. doi: 10.1186/s12938-022-01000-y (PMC9123812; doi:10.1186/s12938-022-01000-y)
Supplement: Supplementary file 3 — Additional file 3: Tab. S1 The numbers of elements and nodes obtained by tetrahedral meshing of models. [file 12938_2022_1000_MOESM3_ESM.docx]

| Model | Number of elements | Number of nodes |
| --- | --- | --- |
| Non-bone graft model | 420922 | 807373 |
| Full maxilla cleft bone graft model | 425533 | 815951 |
| Full alveolar cleft bone graft model | 423210 | 811472 |
| Lower 2/3 bone graft model | 422677 | 810749 |
| Upper 2/3 bone graft model | 422720 | 810819 |
| Lower 1/3 bone graft model | 421996 | 809375 |
| Middle 1/3 bone graft model | 422078 | 809533 |
| Upper 1/3 bone graft model | 422039 | 809445 |
